# Time after Synthesis and Time after Injection Do Not Affect Diagnostic Quality of [^18^F]F-PSMA 1007 PET

**DOI:** 10.3390/cancers14205141

**Published:** 2022-10-20

**Authors:** Elisabeth Relt, Wolfgang Roll, Michael Claesener, Martin Bögemann, Matthias Weckesser, Kambiz Rahbar

**Affiliations:** 1 Department of Nuclear Medicine, University Hospital Münster, 48149 Münster, Germany; 2 West German Cancer Center, 48149 Münster, Germany; 3 Department of Urology, University Hospital Münster, 48149 Münster, Germany

**Keywords:** PSMA-PET, time after synthesis, 18F-PSMA

## Abstract

**Simple Summary:**

The total number of PSMA-PET-CT examinations for the staging of prostate cancer patients has increased in recent years, following its superior imaging properties. Fluorinated PSMA ligands can be produced in larger amounts, facilitating higher patient throughput compared to the initially developed gallium labelled PSMA-tracers; allowing PET in only a few patients per synthesis. This results in a longer time after, typically early morning, radiochemical synthesis (TaS) of [^18^]F-PSMA when the PSMA-PET scan is performed. Moreover, novel fluorinated-PSMA compounds are injected around two hours before PET-CT and the time after injection (TaI) might show significant deviation. [18F]-PSMA PET offers improved detection rates in the proximity of the urinary tract; however, it has the disadvantage of more unspecific uptake, e.g., in the skeleton. This article focusses on the question whether TaS or TaI have an influence on uptake patterns in malignant lesions and healthy tissue.

**Abstract:**

PET imaging using PSMA ligands is increasingly used for staging in prostate cancer patients in different clinical indications. Unlike [^68^Ga]Ga-labeled PSMA ligands, fluorinated compounds can be produced in large amounts; thus, they can be used for a higher number of patients. One concern is that in patients studied a long time after synthesis (TaS) or time after injection (TaI), the specific activity may decline; thus, the signal may be lower in these patients. In this study, we investigated a potential effect of TaS and TaI on image quality. In total, 134 consecutive patients were included in this retrospective analysis on the effect of TaS and TaI on uptake in prostate cancer lesions. All the patients underwent [^18^F]F-PSMA-1007 PET-CT from 99 min up to 549 min after tracer quality control. TaS and TaI were compared to the quantitative tumoral uptake parameters SUVmax and SUVpeak. In a second exploratory part of the analysis, TaS and TaI were correlated to a physiological tracer uptake in different organs. TaS and TaI did not affect the SUVmax and SUVpeak in tumor lesions in [^18^F]F-PSMA-1007 PET. The physiological uptake in salivary glands, lacrimal glands and the ganglia, spleen and urine was not significantly correlated to TaS or TaI; in contrast to the mean liver uptake, showing a weak, but significant correlation to TaS. The [^18^F]F-PSMA-1007 uptake in prostate cancer lesions is not significantly dependent on the TaS and TaI. These results are extremely reassuring when performing [^18^F]F-PSMA-1007 PET a considerable time after synthesis.

## 1. Introduction

The management of primary and recurrent prostate cancer is challenging [1]. Prostate specific membrane antigen (PSMA) positron emission tomography (PET) has been shown to be an excellent imaging method for the detection and delineation of prostate cancer and its metastases [2,3]. Although the name of the substance suggests specific prostate cancer uptake, especially salivary and lacrimal glands, liver and ganglions are present with a high physiological uptake [4,5].

The first experiences with this tracer have been collected using [^68^Ga]Ga-labelled compounds [2]. Since the labelling of PSMA with [^68^Ga]Ga is usually limited to less than 1 GBq, only two to three patients can be studied with a single synthesis. To overcome this limitation, [^18^F]F labelled tracers have been introduced, allowing more patients to be studied with a single synthesis [5,6].

[^18^F]F-PSMA-1007 is one of the most promising compounds showing a high lesion detectability and the possibility to perform imaging as late as two hours after injection [3,5,7]. This is particularly advantageous since after two hours, only minimal tracer uptake in the urine is present; thus, facilitating the diagnosis of local recurrence and of lymph node metastases adjacent to the ureter [5]. On the other hand, this tracer may show high liver uptake due to hepatic metabolism, which may be disadvantageous in the case of liver metastases [8]. Although tracer stability is high [6], free [^18^F]F, a higher proportion of unlabelled substance or a small molar activity might limit the diagnostic accuracy of this tracer at later time points. There is only one study addressing the effect of specific activity on PSMA uptake so far [9]. In that study, only minor effects of specific activity were reported over a broad range of specific activities in another [^18^F]F labelled compound targeting PSMA [9].

The aim of this study was to find out whether a significant correlation of tumour uptake and time after synthesis (TaS) of [^18^F]F-PSMA-1007 or time after injection (TaI) can be found. To complete the analysis, uptake in non-target organs, such as salivary glands, liver, spleen and bladder was assessed as a subanalysis. Furthermore, we also documented diffuse bone uptake, which might occur owing to free [^18^F]F. Specific activity was calculated and maximal uptake intensity was compared for syntheses with lower, intermediate and high specific activity.

## 2. Materials and Methods

### 2.1. Patients

Between February 2020 and the end of May 2020, 134 consecutive prostate cancer patients (median age 70 y, range: 51–82 y) underwent [^18^F]F-PSMA-1007 PET-CT. Most of the patients were studied to detect the source of elevated PSA in the case of biochemical recurrence (70.7 %), in 21.1% and to evaluate the suitability for [177Lu]Lu-PSMA-therapy Additional clinical characteristics are shown in Table 1.

In a second explorative analysis part, we wanted to analyse the influence of specific activity on PET uptake values. Data on specific activity was available in 116 of 134 patients. The maximal and peak standard uptake value (SUV) in the hottest tumour lesion were compared between patients with low (0–1.5 GBq/µg, intermediate (1.5–3.0 GBq/µg) and high (3.0 GBq/µg) specific activity.

### 2.2. Radiotracer and PSMA PET Imaging

The synthesis of [^18^F]F-PSMA-1007 was performed according to a previously published procedure [10]. Image acquisition was started approximately 120 min after intravenous tracer administration. Specific activity of [^18^F]F-PSMA-1007 was determined by analytical HPLC. The determined values ranged between 0.78 and 9.07 GBq/µg. The average value of the obtained specific activity was calculated to be 2.57 GBq/µg after the end of synthesis. A Biograph mCT was used for image acquisition (Siemens Healthineers, Knoxville, TN, USA). PET reconstruction was performed using manufacturer standard tools (with iterative reconstruction and time of flight correction, but without point spread function adjustments), using a whole body low dose CT for attenuation correction.

### 2.3. PET Image Analysis

The images were analysed by using Syngovia software version 27 (Siemens Healthineers, Erlangen, Germany). PET datasets were reviewed by two nuclear medicine specialists and graded as PET positive or PET negative. To date, there is no reliable standard cutoff in SUV defining PET positive and negative lesions. Therefore, readers relied on current guidelines on PSMA-PET assessment [11] and defined the lesions as benign or malignant in consensus. Readers assessed the number of metastases on a per patient basis in predefined groups: 0: no metastases; 1: one metastasis; 2: 2–6 metastases; and 3: >= 7 metastases. SUV were measured using isocontour volumes of interest. Both the voxel with the most intense SUV (SUVmax) and the highest SUV within a region of 1 cm3 (SUVpeak) were measured for each patient using the most intense tumour lesion visible on PET (metastasis or local recurrence/primary tumour). Furthermore, a more subtle analysis was performed using the different potential sites of recurrence (i.e., local recurrence, locoregional lymph nodes, retroperitoneal lymph nodes, extraabdominal lymph nodes, bone metastases, liver metastasis and other sites of metastasis; e.g., adrenal metastases). For all these regions, SUVmax and SUVpeak were documented. Furthermore, the same SUV were also documented for salivary glands and lacrimal glands and for stellate ganglia, for coeliac ganglia and for presacral ganglia (if any of those were detectable). If the ganglia were not detectable, we also tried to find out whether they were undetectable owing to surrounding metastatic activity (that is, locoregional lymph nodes in the case of presacral ganglia, retroperitoneal metastatic activity or bowel activity in the case of coeliac ganglia and bone metastases in the case of stellate ganglia). Additionally, we also documented the mean SUV in the liver, spleen (representative circular ROI) and in the urine (a bladder region defined in the low dose CT).

We also assessed any diffuse bone uptake, which can be detected with this tracer; however, it is of insignificant meaning, There is an ongoing discussion as to whether this occurs due to free fluorine, a bone seeking agent and thus, might be present in “late” patients rather than in “early” patients. Another theory is that diffuse bone uptake due to free fluorine is seen in certain syntheses only and might thus, be clustered in all the patients of a given synthesis.

### 2.4. Statistics

The quantitative PET values SUVmax, SUVpeak and SUVmean are expressed as mean and standard deviation and median with a corresponding range. Clinical variables are presented as mean and standard deviation if normally distributed and as median with a corresponding range if not. Categorial variables are presented as absolute and relative frequencies.

Spearman’s correlation coefficient r was used to measure the strengths of association between the non-normally distributed variables. 

In the primary analysis, TaS and TaI were correlated with the quantitative uptake parameters SUVpeak and SUVmax of PSMA-positive primary tumour/local recurrence and metastases. For the primary analysis, following the Bonferroni-Holm correction for multiple comparisons, results with a *p* < 0.00125 (0.05/40) were regarded as statistically significant.

In the secondary explorative part of the analysis, TaS and TaI were correlated with physiological uptake (SUVmax, SUVpeak) in different organs. The Mann–Whitney U test was used for comparison of the two unmatched groups that are non-normally distributed. For more than two groups, the Kruskal-Wallis test was applied. In exploratory analysis, *p* < 0.05 was regarded as statistically significant. The analysis was performed using SPSS (version 27.0; IBM SPSS, Somers, NY, USA).

## 3. Results

The mean weight of the patients was 88.6 ± 5.2 kg (range: 56–160 kg). The median injected activity was 3.04 MBq/kg bodyweight (range: 2.3 to 4.3 MBq/kg). Measurements were performed between 98 and 211 min after injection and between 99 and 549 min after synthesis.

The overall potential local recurrence or potential metastases were detected in 93.6% of the patients with the following distribution: a single metastasis was found in 19.2%, 2–6 metastases were present in 44.8%, and more than 7 metastases were detected in 36.0% of the patients. In each patient, the highest SUVmax in a potential metastatic site was documented and the highest SUVmax of different sites was documented (local recurrence, locoregional lymph nodes, inguinal and parailiacal, retroperitoneal lymph nodes, extraabdominal lymph nodes, bone metastases, lung metastases, liver metastases and adrenal metastases). We did not establish a histopathological confirmation since this was not necessary for the scope of this study. We also did not calculate any values for sensitivity and specificity for this reason.

Correlative analysis of PSMA-uptake assessed by SUVmax or SUVpeak in local recurrence/primary tumour and metastases revealed no significant correlation with TaS (Table 2). This was also true for the correlation of TaI and the SUVmax or SUVpeak of tumoural lesions (Table 2). The correlation of physiological organ uptake to TaS revealed a weak but significant correlation for the liver SUVmean only with *p* = 0.037 and r = −0.180 (Table 3).

The PSA values did not show a significant correlation with SUVmax (r = 0.15, *p* = ns) or SUVpeak (r = 0.19, *p* = ns); however, they were significantly different in the groups with a different number of metastases (one or no metastases, mean PSA 1.5; and 2 or more metastases, mean PSA 150.2; *p* < 0.01).

The mean TaS was not significantly different in patients with and without the presence of potentially nonspecific bone uptake in PET (mean TaS in positive cases was 268 min, mean TaS in negative cases was 253 min, p = ns). These findings make a correction of the results from Table 2 for the corresponding covariates unnecessary.

An interesting observation was the fact that SUVmax was significantly higher in the coeliac ganglia 5.73 ± 2.25 than in the stellate ganglia (4.28 ± 1.41) (*p* < 0.01) and in the presacral ganglia (3.15 ± 1.78) (*p* < 0.01). Detectability of the ganglia was hampered in the case of adjacent lymph nodes or in case of high bone uptake for the presacral and the stellate ganglia. The coeliac ganglia were harder to detect in the case of bowel activity and in the case of adjacent lymph nodes.

**Figure 1:**
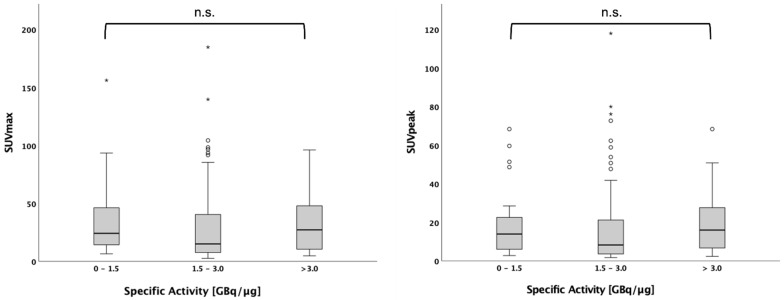
SUVmax and SUVpeak do not depend on specific activity. SUVmax and SUVpeak of the hottest tumor lesion, represented in boxplots with outliers, are not significantly different between patients injected with [^18^F]F PSMA 1007 with a specific activity between 0–1.5 GBq/µg, 1.5–3.0 GBq/µg and above 3.0 GBq/µg.

In n = 116 patients, the data on specific activity were available. The number of patients studied with the activity from a single synthesis was between 4 and 12 patients. Specific activity of the syntheses ranged from 0.78 to 9.07 GBq/µg. Within this range, no significant effect on SUVmax and SUVpeak of the hottest lesion could be found for those patients with a specific activity of 0–1.5 GBq/µg (n = 27), 1.5–3.0 GBq/µg (n = 63) and above 3.0 GBq/µg (n = 26) (*p* = n.s. for both) (Figure 1).

## 4. Discussion

In the present study, there was no major influence of the TaS on tumour uptake. Since the number of patients was high and the distribution of TaS values was broad, this result is quite reassuring when performing [^18^F]F-PSMA-1007 PET.

When it comes to the longitudinal evaluation of SUVmax at two time points (e.g., after therapy and when a high sensitivity is needed, such as in patients with a low but significant PSA recurrence,) the fact that the range of specific activity has no major effect on quantitative uptake values increases reliability. This is at least true for the range of specific activities in the present study, although it is possible that in case of other ranges of specific activity, there may be an influence on the intensity of uptake [10].

We did not find any differences in TaS in those patients who showed unspecific uptake in the bone and those who did not. These “insignificant bone lesions” have low activity and are found in the absence of other significant bone lesions. Patients with these lesions do not have an additional survival risk [12,13]. However, this unspecific uptake might have influence on the cost effectiveness of [^18^F]F-PSMA PET due to necessary additional morphological imaging (e.g., MRI) compared to [^68^Ga]Ga-PSMA PET [14].

In this study, there was a significant negative correlation between the SUVmean of the liver and TaS. However, this correlation was weak. Moreover, there was no significant correlation between physiological uptake in other organs and TaS or TaI.

Another observation, which is in line with the literature, is that PSA values were clearly associated with the number of metastases and thus, to tumor load. In previous studies, this correlation was significant; however, it was mainly moderate [15,16]. Previously published results on a potential correlation between the quantitative PET uptake values SUVmax and SUVpeak are contradictory. In locoregional disease, a significant however moderate correlation was shown in patients before definite radiotherapy [17], in contrast to our results in a mixed cohort of prostate cancer patients.

We did not try to find histopathological confirmation. However, this might not be necessary, since the objective of this study was neither to calculate the diagnostic accuracy of PET, nor to assess the reasons for insignificant bone uptake. It might be advantageous to know whether the accuracy depends on the TaS; however, it appears unlikely as we did not find any hint on the TaS having any influence at all on [^18^F]F-PSMA uptake in primary tumor, local recurrence or prostate cancer metastases. This was, furthermore, not a clinically homogeneous group of patients as to all clinical parameters, hampering a potential calculation of accuracy. However, the advantage of analysing consecutive patients is that they are representative of the spectrum of patients referred for a [^18^F]F-PSMA-PET at a university hospital. Moreover, in the present cohort, the numbers of lung-, liver- and adrenal gland metastases are low, hampering correlative analysis.

A major limitation is the retrospective nature of the presented analysis. In order to really prove that there is no effect of TaS or specific activity, the same patients should be studied on consecutive days and with higher or lower specific activities. Such a study, however, is not justified by the present retrospective data in this sufficiently powered analysis.

## 5. Conclusions

In the present study, TaS and TaI did not influence [^18^F]F-PSMA-1007 uptake in prostate cancer lesions. The high number of patients and the broad distribution of TaS values make these results quite reassuring when performing [^18^F]F-PSMA-1007 PET.

## Figures and Tables

**Table 1:** Characteristics of the 134 patients.

**Parameters**	**Values**
	**Mean ± SD**
Age [y]	70 ± 7.8
PSA [ng/mL] (n = 124)	110.4 ± 390.0
SUVmax (n = 133)	34.9 ± 39.3
SUVpeak (n = 133)	19.7 ± 22.7
**Indication for PSMA PET-CT**	**n (%)**
biochemical recurrence	95 (70.7)
initial staging	5 (3.8)
before PSMA therapy	28 (21.1)
control after PSMA therapy	4 (3.0)
restaging	2 (1.5)
**Previous therapies, (n = 134)**	**n (%)**
Prostatectomy	123 (91.8)
Radiotherapy	111 (82.8)
Androgen deprivation therapy	115 (85.8)
Chemotherapy	101 (75.4)
**Gleason Score (n = 99)**	**n (%)**
4	1 (1.0)
6	5 (5.1)
7	38 (38.4)
8	16 (16.2)
9	34 (34.3)
10	5 (5.1)
**Metastases (n = 125)**	**n (%)**
≤1	24 (19.2)
2–6	56 (44.8)
≥7	45 (36.0)
**Injected activity [MBq/kg]**	**Median (range)**
	3.04 (2.3 to 4.3)

**Table 2:** Correlation of quantitative PSMA-PET parameters of malignant lesions and time after synthesis and time after injection in the primary cohort of 134 consecutive patients.

	Time after Synthesis		Time after Injection	
Location	Spearman Coefficient	*p*-Value	Spearman Coefficient	*p*-Value
SUVmax all (n = 133)	−0.006	0.948	−0.026	0.766
SUVpeak all (n = 133)	−0.005	0.956	−0.036	0.680
Prostate SUVmax (n = 53)	−0.157	0.262	−0.011	0.937
Prostate SUVpeak (n = 53)	−0.103	0.461	0.028	0.843
LN inguinal SUVmax (n = 41)	−0.196	0.830	−0.175	0.273
LN inguinal SUVpeak (n = 41)	−0.035	0.830	−0.042	0.796
LN parailiacal SUVmax (n = 70)	−0.090	0.460	−0.043	0.726
LN parailiacal SUVpeak (n = 70)	−0.095	0.435	−0.012	0.922
LN retroperitoneal SUVmax (n = 35)	−0.019	0.915	−0.197	0.256
LN retroperitoneal SUVpeak (n = 35)	−0.053	0.765	−0.202	0.252
LN extrabdominal SUVmax (n = 52)	−0.066	0.640	0.004	0.978
LN extrabdominal SUVpeak (n = 52)	−0.023	0.871	−0.007	0.960
Bone SUVmax (n = 97)	0.102	0.321	0.076	0.458
Bone SUVpeak (n = 97)	0.103	0.318	0.080	0.437
Lung SUVmax (n = 12)	−0.273	0.390	0.126	0.696
Lung SUVpeak (n = 12)	−0.273	0.390	0.126	0.696
Liver SUVmax (n = 7)	0.571	0.180	0.214	0.610
Liver SUVpeak (n = 7)	0.643	0.119	0.286	0.493
Adrenal Gland SUVmax (n = 11)	0.491	0.125	0.140	0.665
Adrenal Gland SUVpeak (n = 11)	0.400	0.223	0.077	0.812

**Table 3:** Correlation of quantitative PSMA-PET parameters of different organs with known elevated physiological uptake and time after synthesis/time after injection.

Location		Time after Synthesis	Time after Injection
Liver SUVmean	Spearman coefficient	−0.180	−0.045
	*p*-value	0.037	0.607
Spleen SUVmean	Spearman coefficient	0.157	−0.069
	*p*-value	0.070	0.425
Urine SUVmean	Spearman coefficient	0.076	0.038
	*p*-value	0.385	0.663
Salivary Gland SUVmax	Spearman coefficient	−0.105	−0.024
	*p*-value	0.228	0.784
Salivary Gland SUVpeak	Spearman coefficient	−0.134	−0.045
	*p*-value	0.122	0.608
Lacrimal Gland SUVmax	Spearman coefficient	−0.014	−0.038
	*p*-value	0.870	0.662
Lacrimal Gland SUVpeak	Spearman coefficient	−0.075	−0.062
	*p*-value	0.390	0.476
Stellate ganglione SUVmax	Spearman coefficient	−0.035	−0.036
	*p*-value	0.732	0.719
Stellate ganglione SUVpeak	Spearman coefficient	−0.075	−0.075
	*p*-value	0.459	0.456
Coeliac ganglion SUVmax	Spearman coefficient	−0.033	−0.063
	*p*-value	0.736	0.517
Coeliac ganglion SUVpeak	Spearman coefficient	0.000	−0.107
	*p*-value	0.996	0.266
Sacral ganglion SUVmax	Spearman coefficient	−0.114	−0.015
	*p*-value	0.349	0.903
Sacral ganglion SUVpeak	Spearman coefficient	−0.215	−0.095
	*p*-value	0.074	0.435

## Data Availability

Data are contained within the article.

## References

[1] Sartor O., de Bono J.S. (2018). Metastatic Prostate Cancer. N. Engl. J. Med..

[2] Afshar-Oromieh A., Holland-Letz T., Giesel F.L., Kratochwil C., Mier W., Haufe S., Debus N., Eder M., Eisenhut M., Schäfer M. (2017). Diagnostic performance of (68)Ga-PSMA-11 (HBED-CC) PET/CT in patients with recurrent prostate cancer: Evaluation in 1007 patients. Eur. J. Nucl. Med. Mol. Imaging.

[3] Rahbar K., Afshar-Oromieh A., Seifert R., Wagner S., Schäfers M., Bögemann M., Weckesser M. (2018). Diagnostic performance of (18)F-PSMA-1007 PET/CT in patients with biochemical recurrent prostate cancer. Eur. J. Nucl. Med. Mol. Imaging.

[4] Backhaus P., Noto B., Avramovic N., Grubert L.S., Huss S., Bögemann M., Stegger L., Weckesser M., Schäfers M., Rahbar K. (2018). Targeting PSMA by radioligands in non-prostate disease-current status and future perspectives. Eur. J. Nucl. Med. Mol. Imaging.

[5] Giesel F.L., Hadaschik B., Cardinale J., Radtke J., Vinsensia M., Lehnert W., Kesch C., Tolstov Y., Singer S., Grabe N. (2017). F-18 labelled PSMA-1007: Biodistribution, radiation dosimetry and histopathological validation of tumor lesions in prostate cancer patients. Eur. J. Nucl. Med. Mol. Imaging.

[6] Giesel F.L., Cardinale J., Schäfer M., Neels O., Benešová M., Mier W., Haberkorn U., Kopka K., Kratochwil C. (2016). (18)F-Labelled PSMA-1007 shows similarity in structure, biodistribution and tumour uptake to the theragnostic compound PSMA-617. Eur. J. Nucl. Med. Mol. Imaging.

[7] Giesel F.L., Knorr K., Spohn F., Will L., Maurer T., Flechsig P., Neels O., Schiller K., Amaral H., Weber W.A. (2019). Detection Efficacy of (18)F-PSMA-1007 PET/CT in 251 Patients with Biochemical Recurrence of Prostate Cancer after Radical Prostatectomy. J. Nucl. Med..

[8] Rauscher I., Krönke M., König M., Gafita A., Maurer T., Horn T., Schiller K., Weber W., Eiber M. (2020). Matched-Pair Comparison of (68)Ga-PSMA-11 PET/CT and (18)F-PSMA-1007 PET/CT: Frequency of Pitfalls and Detection Efficacy in Biochemical Recurrence after Radical Prostatectomy. J. Nucl. Med..

[9] Langbein T., Wurzer A., Gafita A., Robertson A., Wang H., Arcay A., Herz M., Wester H.-J., Weber W.A., Eiber M. (2021). The Influence of Specific Activity on the Biodistribution of (18)F-rhPSMA-7.3: A Retrospective Analysis of Clinical Positron Emission Tomography Data. J. Nucl. Med..

[10] Cardinale J., Martin R., Remde Y., Schäfer M., Hienzsch A., Hübner S., Zerges A.-M., Marx H., Hesse R., Weber K. (2017). Procedures for the GMP-Compliant Production and Quality Control of [(18)F]PSMA-1007: A Next Generation Radiofluorinated Tracer for the Detection of Prostate Cancer. Pharmaceuticals.

[11] Rowe S.P., Pienta K.J., Pomper M.G., Gorin M.A. (2018). PSMA-RADS Version 1.0: A Step Towards Standardizing the Interpretation and Reporting of PSMA-targeted PET Imaging Studies. Eur. Urol..

[12] Grünig H., Maurer A., Thali Y., Kovacs Z., Strobel K., Burger I.A., Müller J. (2021). Focal unspecific bone uptake on [(18)F]-PSMA-1007 PET: A multicenter retrospective evaluation of the distribution, frequency, and quantitative parameters of a potential pitfall in prostate cancer imaging. Eur. J. Nucl. Med. Mol. Imaging.

[13] Arnfield E.G., Thomas P.A., Roberts M.J., Pelecanos A.M., Ramsay S.C., Lin C.Y., Latter M.J., Garcia P.L., Pattison D.A. (2021). Clinical insignificance of [(18)F]PSMA-1007 avid non-specific bone lesions: A retrospective evaluation. Eur. J. Nucl. Med. Mol. Imaging.

[14] Alberts I., Mingels C., Zacho H.D., Lanz S., Schöder H., Rominger A., Zwahlen M., Afshar-Oromieh A. (2021). Comparing the clinical performance and cost efficacy of [68Ga]Ga-PSMA-11 and [18F]PSMA-1007 in the diagnosis of recurrent prostate cancer: A Markov chain decision analysis. Eur. J. Nucl. Med. Mol. Imaging.

[15] Seifert R., Herrmann K., Kleesiek J., Schäfers M., Shah V., Xu Z., Chabin G., Grbic S., Spottiswoode B., Rahbar K. (2020). Semiautomatically Quantified Tumor Volume Using 68Ga-PSMA-11 PET as a Biomarker for Survival in Patients with Advanced Prostate Cancer. J. Nucl. Med..

[16] Santos A., Mattiolli A., Carvalheira J.B., Ferreira U., Camacho M., Silva C., Costa F., Matheus W., Lima M., Etchebehere E. (2021). PSMA whole-body tumor burden in primary staging and biochemical recurrence of prostate cancer. Eur. J. Nucl. Med. Mol. Imaging.

[17] Onal C., Torun N., Oymak E., Guler O.C., Reyhan M., Yapar A.F. (2020). Retrospective correlation of (68)ga-psma uptake with clinical parameters in prostate cancer patients undergoing definitive radiotherapy. Ann. Nucl. Med..

